# Supplementary L-arginine can enhance reproductive parameters and outcomes in large mammals

**DOI:** 10.3389/fvets.2025.1740399

**Published:** 2026-01-12

**Authors:** Megan M. Ohrt, Nancy H. Ing

**Affiliations:** Department of Animal Science, Texas A&M University, College Station, TX, United States

**Keywords:** dietary supplement, fertility, heat stress, pregnancy, semen quality, spermatozoa

## Abstract

The amino acid L-arginine (Arg) is not only proteinogenic, but also a powerful regulator of cell physiology. Arg activates the mechanistic target of rapamycin directly, which regulates numerous kinase pathways. Arg also is metabolized to nitric oxide (a powerful cell signaling molecule) and polyamines, which stabilize proteins and DNA structurally. This Mini Review focusses on the effects of dietary Arg supplementation on reproductive parameters and outcomes in large mammalian species. Studies of Arg supplementation demonstrate consistent benefits to pregnancies in females and sperm quality in male livestock and men. We also present a summary of the numerous and rapid beneficial effects of *in vitro* Arg supplementation on sperm quality. Dietary and *in vitro* Arg also appear to reduce the damage to sperm caused by heat stress. However, there is an absence of Arg studies in stallions and dogs: two species that have substantial assisted reproductive technology done by veterinarians and others. Overall, Arg appears to be a safe, inexpensive, and natural supplement that is useful for improving reproductive outcomes in mammals.

## 1 Introduction

L-arginine (Arg) is a dietary amino acid that is, like others, incorporated into proteins. But perhaps more importantly, Arg has major roles in the regulation of metabolism. While Arg affects most physiological systems in mammals, its effects on reproduction are remarkable and have far-reaching consequences. Our objective is to review the multiple functions of Arg on physiology and the dietary supplementation studies that discovered beneficial effects on reproduction in livestock and human species. In addition, rapid effects on sperm *in vitro* are presented. Importantly, Arg supplementation *in vivo* and *in vitro* appears to protect sperm from damage induced by heat stress. Arg supplementation may provide one way that veterinary science professionals can use to enhance the reproductive efficiency of domestic animals around the world. For livestock species, this would make production more sustainable and ensure that high quality protein sources are available for our growing human population.

### 1.1 L-arginine (Arg) supplementation affects many physiological systems

Arg is one of the 20 amino acids that are the building blocks of animal proteins. In addition, Arg and its metabolites have several effects that regulate cell processes. For most mammals, Arg was traditionally classified as a nutritionally non-essential amino acid because the enterocytes of the intestine synthesize it. However, some species, including horses and cats *do not* synthesize Arg *de novo* (1, 2); thus, it is a nutritionally essential amino acid for them and is required in their diets. In addition to being proteinogenic, Arg is one of six “functional amino acids” that have major roles as regulators of key metabolic pathways (3). For example, Arg directly activates the mechanistic Target of Rapamycin (mTOR, a powerful protein kinase) pathway to increase protein synthesis. The Arg metabolites nitric oxide (NO) and polyamines are also very bioactive. Arg is metabolized by NO synthases to NO, which is a vasodilator, neurotransmitter, and signaling molecule involved in angiogenesis, regulation of cell metabolism, and apoptosis. Arg is also metabolized to polyamines: putrescine, spermine, and spermidine, which have central roles in gene expression, DNA and protein synthesis, and cell proliferation and differentiation (4, 5). Furthermore, dietary Arg supplementation improved cardiovascular and endothelial function in rats and humans (5). It also had positive effects on immune, muscle, brain function, metabolism, and tissue repair. In young men under stressful conditions, Arg supplementation enhanced neuroendocrine and cardiovascular health as well as mood (6). In neonatal pigs, Arg supplementation improved intestinal health and added lean muscle (7). In mice, Arg supplementation altered intestinal microbiota to increase beneficial bacteria (8). Together, these examples demonstrate that dietary Arg supplementation has remarkably positive effects on numerous physiological systems in mammals.

### 1.2 Dietary Arg supplementation is safe

Arg supplementation has been tested in numerous animal species and is generally safe with very few side effects (7). Arg is a component of normal diets, with humans eating up to 5 g daily (9). In humans, supplementation with very high doses of Arg [300 mg/kg body weight (BW)*day = 25 g per day for someone weighing 83 kg or 183 lbs.] is considered safe (10, 11). The effective doses in women and men are much lower, ranging from 3 to 14 g Arg per day for 1 to 4 weeks (Tables 1, 2). The only slightly deleterious effect of feeding Arg was found in mares fed 250 mg/kg BW (12). That Arg dosage interfered with the intestinal absorption of lysine and methionine, thereby decreasing serum concentrations of those two essential amino acids by 43 and 29%, respectively, at 2 h post-feeding. The lower dose of 125 mg Arg/kg BW in that study raised serum Arg concentrations by 240% without affecting serum lysine or methionine concentrations. Studies of pigs (13) and rats (14) indicate that long-term (90 days) dietary supplementation of <2% Arg in the diet did not result in any adverse effects.

**Table 1:** Arg supplementation in the diets of pregnant mammals improves reproductive outcomes.

Arg supplementation effects	Species	Daily Arg dose	Duration	Reference
↑ Fetal well-being	Woman with PE	3 g	3 weeks	(57)
↓ Blood pressure	Woman with PE	14 g	1 week	(58)
↑ Fetal growth	Woman with PE	3 g	3 weeks	(59)
↓ PE symptoms	Woman with PE risk	6.6 g	8–26 weeks	(60)
↑ Litter size & birthweights	Pig	120 mg/kg BW	11 weeks	(26)
↑ Fetal weight & placental function	Pig	80 mg/kg BW	2 weeks	(27)
↑ Embryo/fetal size	Mare	125 mg/kg BW	4 weeks	(29)
**↓** Insulin resistance	Mare	192 mg/kg BW	19 weeks	(31)
↑ Uterine artery blood flow	Mare	50 mg/kg BW	47 weeks	(30)

Studies are listed in chronological order within a species. “PE” is pre-eclampsia, a serious hypertensive disease of pregnant women. “BW” is body weight.

**Table 2:** Arg supplementation in the diets of male mammals improves semen parameters.

Arg increased	Species	Daily Arg dose	Earliest effect	Reference
↑ NUM & MOT	Man (subfertile)	10 g	1–3 weeks	(4)
↑ NUM & MOT	Man	0.5 g	2 weeks	(9)
↑ MOT	Man (subfertile)	8 g	4 weeks	(32)
↑ NUM & MOT	Man	3 g	4 weeks	(35)
↑ NUM & MOT	Boar	100 mg/kg BW	4.3 weeks	(3)
↑ NUM, MOT, & MORPH	Boar	60, 80, 100 mg/kg BW	12 weeks	(38)
↑ NUM & MOT	Ram	5 mg/kg BW (1 injection)	1–5 days	(39)
↑ NUM, MOT, & MORPH	Ram	20 mg/kg BW	4.3 weeks	(37)

Each of the studies (in chronological order within a species) identified one or more improved parameters: sperm number (NUM), % total motility (MOT), and % normal morphology (MORPH) in response to Arg supplementation. The exception to dietary supplementation (intraperitoneal injection) is underlined. Experimental subjects were normal adult males except where noted as “subfertile”.

### 1.3 Heat stress impairs semen quality and male fertility

Heat stress is occurring more frequently due to global warming, and it is affecting many temperate regions. The year 2024 was the warmest across the globe since record-keeping began in 1850 (15). Livestock producers worldwide are searching for strategies to mitigate heat stress (16, 17). The testis is the most heat-sensitive organ in mammals (18). In the scrotum, the testes reside at 33–35 °C. Heat stress in male livestock, including bulls, rams, and boars, has negative effects on sperm production, including decreased numbers, motility, and normal morphology as well as fertility (16, 18, 19). Even modest heat stress can have profound effects. For example, increasing the scrotal temperatures of rams by 1.2 °C for 12 h on four consecutive days resulted in severe decreases in sperm quality (20). 24 days later, sperm numbers were 30% lower, total motility was 0%, only 6% of sperm had normal morphology and 0% had fully intact DNA. Heat-stressed stallions suffer from subfertility, poor semen quality, and low serum testosterone (16, 21–24). Novel methods to reduce sperm damage from heat stress are needed.

## 2 Arg supplementation enhances reproductive parameters

### 2.1 Dietary Arg supplementation of pregnant females improves reproductive outcomes

Arg supplementation has many different positive effects on female reproduction (Table 1). Arg supplementation is used to treat serious hypertensive illnesses in pregnant women such as pre-eclampsia (5, 25). Female pigs and rats supplemented with Arg had more offspring and they had greater birthweights (26). Young female pigs supplemented between days 14 and 30 of the 112 day-long gestation period had increased embryonic survival, fetal weights, and placental vasculature and function (27). In pigs, Arg supplementation enhanced oocyte quality, embryo survival, fetal health, and placental function (5). Arg supplementation (100 mg Arg/kg BW*day) by intravenous injection increased serum prolactin and subsequent lactation in dairy cows (28). Given to pregnant mares, Arg doses ranging from 50 to 192 mg/kg BW*day increased embryo and fetal size, and blood flow in the uterine arteries, while it decreased insulin resistance (29–31, 61). Overall, Arg supplementation has tremendous potential to augment reproductive success in female mammals.

### 2.2 Dietary Arg supplementation improves reproductive parameters in males

In men, the effects of Arg supplementation on semen quality have been studied for eight decades and include increased sperm numbers, motility, and normal morphology (Table 2). Since 1944, dietary Arg has been known to be required for spermatogenesis in men and rats (4), and it is likely to be true for all mammals. In that study, three men were given a diet lacking Arg. Semen collected 9 days later showed 88% lower sperm counts and 95% lower motile sperm. The men were immediately placed on a normal diet and recovered to have normal semen parameters within 3 weeks. The Holt and Albanese study also reported that feeding male rats an Arg-deficient diet for 47 days destroyed the histoarchitecture of the seminiferous tubules, within which the male germ cells develop over 2 months. When Holt & Albanese (4) supplemented men with low sperm counts (oligospermia) with 122 mg Arg/kg BW*day, they detected increases in both sperm numbers (250%) and motility (100%) within 2 weeks. Tanimura (9) reported that supplementing men with 7 mg Arg/kg BW*day increased sperm counts by 60% within 2 weeks. Forty men with low percentages of motile sperm (asthenospermia) improved with 40 mg Arg/kg BW*day within 4 weeks (32). Both Arg metabolites NO and polyamines play important roles in spermatogenesis (33, 34). Arg supplementation of men also enhanced libido and is now used to treat erectile dysfunction: a daily supplement named Prelox® is marketed worldwide and contains 3 g Arg (35). These rapid and profound effects of Arg supplementation of men lead to studies in livestock.

Livestock studies with Arg supplementation identified the effects on semen that were seen in men (Table 2), as well as increased chromatin condensation in sperm heads and serum testosterone. Arg supplementation of boars (100 mg/kg BW*day) enhanced semen quality, including increased sperm counts (3). Testosterone is involved in many aspects of spermatogenesis (36). Testosterone drives chromatin condensation in late spermatogenesis when protamine proteins (made up of 50% Arg) replace 85% of histone in chromatin. Arg supplementation increased chromatin condensation in sperm of boars (19%; (37)) and rams (23%; (38)). Note that ruminants can be injected intravenously or intraperitoneally with Arg or fed a coated “rumen-protected” Arg supplement to bypass metabolism by the rumen. In Ozer Kaya et al. (39), rams injected once with 5 mg Arg/kg BW showed increased in sperm numbers (144%) and motility (47%), respectively, within 5 days. Supplementation of rams with rumen-protected Arg for 4 weeks enhanced sperm numbers (166%), motility (11%), sperm with normal morphology (17%), and serum testosterone concentrations (19%), as well as libido (37). These data demonstrate significant semen improvements in short time frames and provide compelling reasons to use Arg supplementation to optimize male reproduction. Notably, although stallions frequently have periods of subfertility (40), Arg supplementation has not yet been tested in them.

### 2.3 *In vitro* Arg supplementation improves sperm quality parameters

*In vitro* studies of sperm from major livestock species have demonstrated that Arg improves specific sperm quality parameters. In a study using goat sperm isolated from the caudal epididymis, 5 mM Arg added to sperm in Sp-TALP medium at 37 °C increased the percentage of total sperm motility by 16%, capacitated sperm by 38% after 4 h of incubation (41). They discovered that the *in vitro* Arg supplementation up-regulated the levels of four mRNAs in sperm that are involved in motility: phosphoglycerate kinase 2 (PGK2), ribonuclease 10 (RNASE10), outer dense fiber of sperm tails 1 (ODF1), and rhophilin associated tail protein 1 like (ROPN1L). Changes in mRNA levels in sperm are likely caused by altered stabilities. The mRNAs in sperm are fragmented to an average length of 250 nucleotides and are unlikely to be translated (42). However, the RNAs that sperm deliver to oocytes at fertilization are critical for embryo development (43). In a study of ejaculated boar sperm, the addition of 1 mM Arg to Ham’s F10 medium before a 1 h of incubation at 39 °C increased the percentage of total sperm motility by 14% and progressively motile sperm by 18% and the 1 mM dose of Arg was more effective than 2 mM (44). It is noteworthy that the percentage of motile sperm in all of these *in vitro* systems decreases over time. While the effects of Arg supplementation are reported as increases in motility at a time point, they may be more correctly stated as preservation of motility or prevention of its loss. Supplementation of boar sperm with 1 mM Arg also decreased levels of cytochrome c oxidase subunit 5B (COX5B) mRNA by 79% and reactive oxygen species (ROS) by 52% while it increased the percentage of sperm with high mitochondrial membrane potential (ΔΨm) by 18% and the activity of mitochondrial respiratory chain complex V (MRCCV) by 33% (44). This implicates Arg in the regulation of mitochondrial oxidative phosphorylation (OXPHOS) on sperm. Ejaculated, frozen and thawed bull sperm were incubated in Sp-TALP lacking or containing 1 mM Arg for 3 h at 38.5° (45). Arg supplementation increased motile sperm by 12%, capacitated sperm by 12%, and sperm with high mitochondrial membrane potential by 20%. Concurrently, proteomic analyses identified 367 proteins, with 11 up-regulated and 29 down-regulated by Arg supplementation. Intriguingly, the A-kinase anchoring protein 3 (AKAP3) was uniquely expressed highly in sperm with Arg supplementation. The ROPN1L mRNA up-regulated by Arg in goat sperm (41) encodes a protein that interacts with AKAP3 protein. In a study performed with ejaculated ram sperm supplementation with 10 mM Arg in complete TALP medium for 3 h at 39 °C, the Arg elevated the percentage of capacitated sperm by 11% and the percentage of acrosome-reacted sperm by 15% compared to controls (46). Coordinately, the percentage of live sperm with high nitric oxide (NO) levels increased by 7%. Based on these studies of goat, boar, bull, and ram sperm, it is apparent that Arg supplementation *in vitro* had positive impacts on several sperm quality parameters including increased motility, NO levels, and ΔΨm, along with decreased ROS, and altered expression of genes involved in motility.

### 2.4 Proteomic studies highlight the importance of Arg and its metabolism in male reproduction

With managers commonly using assisted reproductive technologies with stallions, proteomic studies help lead that methodology (47). A recent publication identified 140 differentially expressed proteins in sperm from fertile versus subfertile stallions that carry a susceptibility genotype (FKBP6 mutation) for impaired acrosome function (48). Stallion sperm parameters, including sperm motility, have related to fertility and differential abundance of specific proteins (49). A study of stallion semen collected during periods of higher and lower fertility identified 38 differentially expressed proteins (40). Proteomic comparisons of bovine caudal epididymal fluid and seminal plasma identified differential expression of proteins within 28 KEGG pathways, including “Arginine and proline metabolism” (50). *In vitro* supplementation of bull sperm with 1 mM Arg for 3 h up-regulated 11 and down-regulated 27 sperm proteins (45). One of the down-regulated proteins was ornithine decarboxylase antizyme 3 (OAZ3), which is prominent within the Arg metabolic pathway. This is consistent with our identification of lower OAZ3 mRNA levels in more dense (more functional) stallion sperm compared to less dense sperm (51).

Studies of sperm proteins that are related to functional characteristics enhance our understanding of gamete physiology. Although sperm do not synthesize proteins, their membranes actively exchange proteins within the epididymis (52). In stallions, sperm spend the last 9 days of the spermatogenic cycle in the epididymis (53). Dietary Arg supplementation increases sperm numbers maximally within days to weeks (4, 39), so it would be plausible their action on sperm in the epididymis to enhance survival and development (54).

### 2.5 Arg supplementation can mitigate the effects of heat stress on male reproduction

There is one study of dietary Arg supplementation of boars before and during summer, improving sperm numbers, motility, and morphology as well as increasing serum testosterone and libido (38). Interestingly, even a single intramuscular injection of 5 mg Arg/kg BW given to rams in severe environmental heat stress increased serum testosterone and NO concentrations and testicular artery blood flow (55). Remarkably, rams that underwent scrotal insulation weeks before semen collection had increased sperm motility, sperm with normal morphology, and intact DNA after freeze-thawing when the extender contained 1 mM Arg (56). The benefits of Arg supplementation of male mice subjected to heat stress include 39% greater progressive motility of sperm and 40% higher serum testosterone (8). Remarkably, transplantation of fecal microbiota from Arg-treated mice enhanced the progressive motility of sperm by 109% and serum testosterone by 24% after heat stress compared to male mice receiving fecal microbiota from mice lacking Arg supplementation. Together, these data reflect on the broad effects of dietary Arg supplementation to promote the health of male reproduction, even in conditions of heat stress.

## 3 Discussion

Arg is a remarkable amino acid for its many functions. Not only is it a building block of proteins, but also it directly activates mTOR and associated kinase pathways. Arg indirectly is involved in cell signaling by being metabolized to NO, which has numerous tissue effects including vasodilation. Arg is also metabolized to polyamines, which play critical roles in stabilizing DNA and protein structures. These mechanisms are involved with the results seen from Arg supplementation in livestock and humans. Dietary supplementation with Arg is safe, economical, easy (usually top-dressing grain), and it is beneficial to reproductive outcomes. Many effects of Arg are rapid, occurring within hours to days. This implicates the cell signaling effects of Arg, among others. In pregnant females, it increased fetal size and birthweights. In males, dietary supplementation of Arg enhanced factors ranging from libido to sperm motility. It is exciting that even addition of Arg to semen extender before freezing increases sperm motility and decreases DNA fragmentation at thawing. The studies that demonstrate the Arg supplementation protects sperm from damage by heat *in vivo* and *in vitro* are additional incentives to use it in promoting reproduction during environmental stressors. Future studies could address the effects of dietary Arg on female breeding success and *in vitro* Arg effects on ova. In addition, the combined effects of dietary and *in vitro* Arg supplementation remain to be investigated in males and females. The future appears bright for using Arg supplementation for enhancing reproductive outcomes in mammals.

## 4 Conclusion

Arg supplementation, dietary and *in vitro*, are examples of how veterinary science can assist livestock producers in improving reproductive efficiency. It joins other approaches by scientists and producers to optimize livestock production. This will make livestock production more sustainable and beneficial for the health of animals, people, and the environment.
